# The endoscopic ultrasound probe findings in prediction of esophageal variceal recurrence after endoscopic variceal eradication therapies in cirrhotic patients: a cohort prospective study

**DOI:** 10.1186/s12876-019-0943-y

**Published:** 2019-02-19

**Authors:** Junfu Zheng, Yuening Zhang, Peng Li, Shibin Zhang, Yue Li, Lei Li, Huiguo Ding

**Affiliations:** Department of Gastroenterology and Hepatology, Beijing You An Hospital affiliated to the Capital Medical University, 8 Xi Tou Tiao, Youanmen wai, Beijing, 100069 China

**Keywords:** Esophageal varice, Liver cirrhosis, Portal hypertension, Endoscopic ultrasound probe examinations

## Abstract

**Background:**

The recurrence of esophageal varices remains high in patients with hepatic portal hypertension after the endoscopic esophageal variceal eradication therapies, including endoscopic variceal band ligation (EVL), injection sclerotherapy (EIS) or EVL plus EIS. The aim of this study was to evaluate the endoscopic ultrasound probe examinations (EUP) findings in the prediction of recurrence following esophageal variceal eradication in a prospective cohort.

**Methods:**

A total of 206 cirrhotic portal hypertension patients with esophageal variceal eradication, who underwent endoscopic variceal therapy (EVL or EIS or EVL plus EIS) were initially enrolled. All patients were scheduled for a follow-up every 6 months for up to 3 years. EUP was performed to evaluate peri-esophageal collateral veins (peri-ECVs), perforating veins (PFV) and para-esophageal collateral veins (para-ECVs). In addition, computed tomography (CT) were conducted to detect portal vein diameter, portal vein embolus, and major portosystemic collateral shunts. The relationship between esophageal variceal recurrence and EUP findings were analyzed.

**Results:**

We found that as high as 93.5% of patients developed esophageal variceal recurrence in the 3-year follow-up. The time of esophageal variceal recurrence after variceal eradication was 13.4 months (13.4 ± 9.2 months). Furthermore, the median time of recurrence in patients who were undertaken EVL,EIS and EVL plus EIS was 10, 13 and 12 months, respectively. We identified that the risk factors, including EVL (OR 0.23, 95% CI 0.08–0.71, *p* < 0.01), Child-Pugh score (OR 3.32,95% CI 1.31–35.35, *p* < 0.05), large peri-ECVs (OR 4.56, 95% CI 2.17–9.58, *p* < 0.0001), and existence of PFV (OR 2.14, 95% CI 1.44–3.16, *p* < 0.001), were significantly associated with the recurrence of esophageal varices. The peri-ECVs and PFV showed better ability to predict esophageal variceal recurrence. When cut-off value of peri-ECVs diameter was 3.5 mm, the specificity of prediction 1-year variceal recurrence was 86% and the sensitivity was 45%.

**Conclusions:**

The EUP appears to be very effective, convenient and economical examinations to predict esophageal varices recurrence after variceal eradication by endoscopic therapies. The high Child-pugh score, large peri-ECVs, and PFV are independent risk factors related to esophageal varices recurrence.

## Background

The esophageal-gastric variceal bleeding (EVB) is considered as one of major fatal complications in patients with hepatic cirrhosis and portal hypertension [1, 2]. Endoscopic variceal ligation (EVL), endoscopic injection-sclerotherapy (EIS), and the two in combination have been recommended by a majority of guidelines for the prevention and treatment of EVB in cirrhotic patients [2, 3]. Notably, the recurrence of esophageal varices is highly prevalent after the endoscopic esophageal variceal eradication therapies in patients with portal hypertension [4, 5]. Although the underlying pathological mechanisms remain to be elucidated, it would be urgent to identify the independent risk factors associated with the recurrence of the esophageal varices as soon as early intervention treatment possible in order to prevent esophageal variceal rebleeding [6].

The endoscopic untrasonography (EUS) has capability to evaluate the blood vessels around the wall of the esophagus in patients with portal hypertension [7]. Recently, It have been reported that the peri-esophageal collateral veins, perforating veins, and para-esophageal collateral veins found by EUS were related to the variceal recurrence following endoscopic therapies [8, 9]. However, these previous studies had small samples, short follow-up period (ranging from 1 to 3 months) and EUS was frequently performed before endoscopic therapies. Currently, the endoscopic ultrasound probe examinations (EUP) is easy and safe to operate compared with EUS performing [10, 11]. However, It was less knowledge that the EUP findings were as same usefulness as EUS in prediction variceal recurrence following endoscopic therapies. Thus, In this prospective cohort study, we aimed to evaluate the EUP findings after the esophageal variceal eradication by EVL, EIS, or EVL plus EIS to predict the variceal recurrence.

## Methods

### Patients

In this prospective cohort, patients who were diagnosed as esophageal varices as well as complicated with liver cirrhosis and portal hypertension were enrolled during the period from January 2012 to December 2014. Of these, a total of 206 patients met the inclusion and exclusion criteria. During follow-up period, 53 individuals were ruled out. Of them, lost or less than 3-year follow-up 23, received TIPS or surgery 9, and died 21. Total 153 patients were enrolled for study. The inclusion criteria were as follows: (1) Age from 18 to 75 years old cirrhotic patients without any treatemt history for portal hypertension, including oral non-selective beta blockers, interventional radiology (such as TIPS), or surgical therapy (splenectomy and devascularization); (2) Esophageal varices were caused by liver cirrhosis with portal hypertension, (3) Esophageal varices were diagnosed by endosopy according to the guidelines [10], (4) The esophageal varices were individually treated by EVL, EIS, or EVL plus EIS. The following exclusion criteria were used in this study: (1) Endoscopic treatment failed to achieve eradication of esophageal varices, (2) TIPS, surgery or death were identified during the 3-year follow-up period, (3) Gastric varices (type GOV2, GOV3) or isolated gastric varices (IGV), (4) Child-Pugh score more than 14, (5) The function failures in the renal, brain, and heart, (6) Esophageal varices caused by no-cirrhotic portal hypertension.

### Endoscopic therapies

**Fig. 1:**
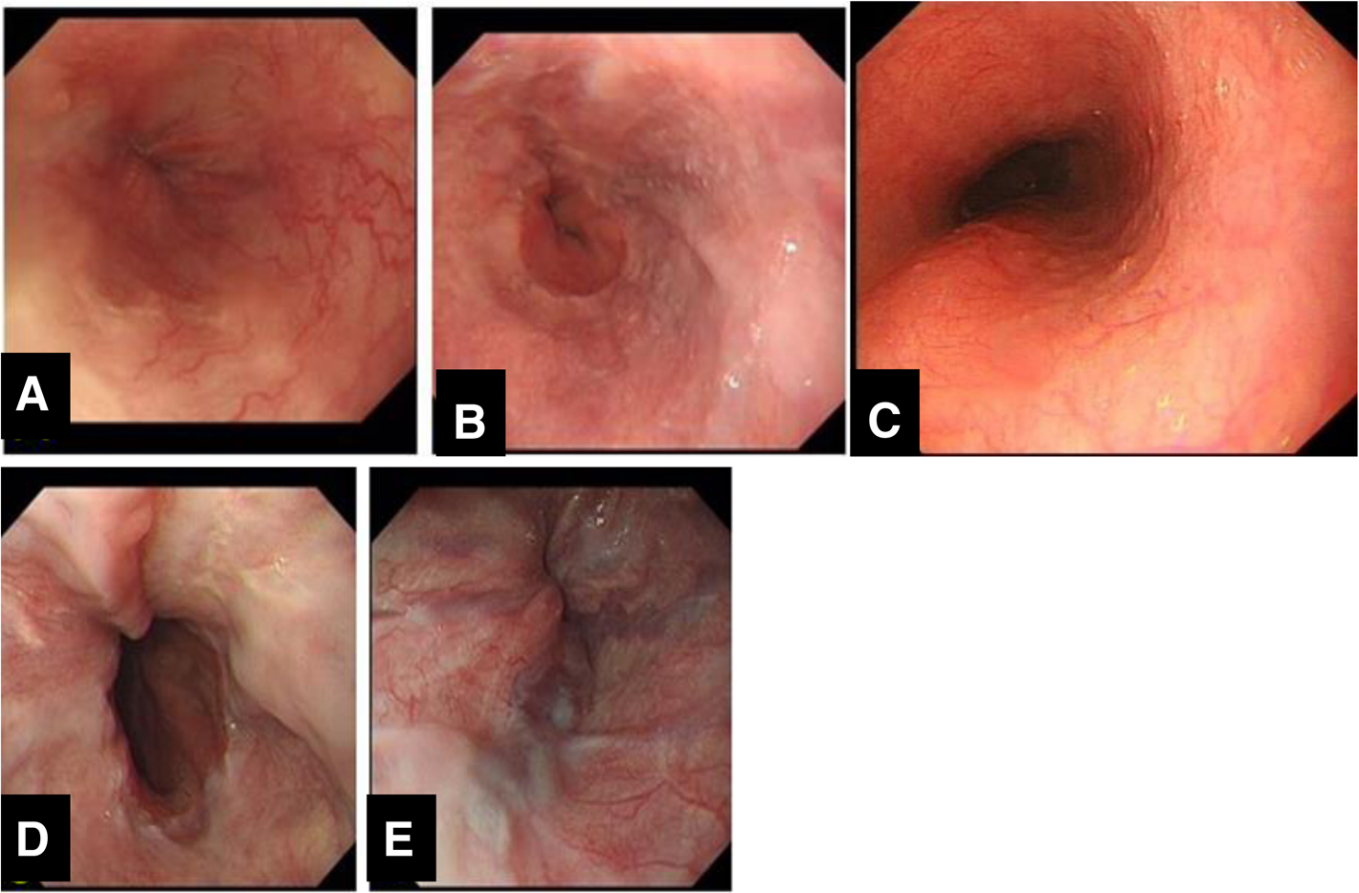
Diagnostic criteria of endoscopic esophageal variceal eradication or recurrences under endoscopy. **a**-**c**:The criteria of endoscopic eradication esophageal varices: lesions lack a varicose (**a**-**b**) or normal esophageal mucosa (**c**). **d**-**e**: The recurrence of esophageal varices:moderate varices(**d**), or mild varicose vein with red sign(**e**)

The flexible GI endoscope (GIF-CV290, Olympus, Japan) was used for diagnosis and treatment for esophageal varices. The methods of endoscopic treatment for esophageal varices included EIS, EVL, and EVL plus EIS. 30–40 mL of 1% lauromacrogol(Xian, China) or 5% sodium morrhuate(Shanghai, China) were used for one course of EIS. The super 7 multiple band ligator (Speedband superview,Boston science, USA) was used for one course of EVL. The endoscopic therapies were carried out by the experienced chief physician. As we known, the superficial varices of the esophageal mucosa are the most important risk factors for bleeding. Therefore, the variceal eradication were defined as endoscopic normal esophageal mucosa or lesions lack a varicose appearance of the esophageal mucosa after one or more courses of endoscopic treatments (Fig. 1a-c).

### Endoscopic ultrasound probe examinations(EUP)

**Fig. 2:**
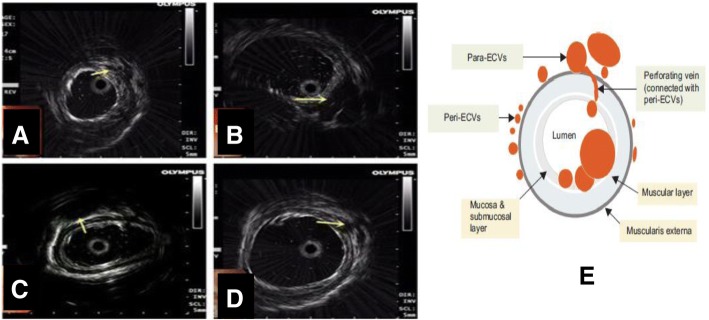
Endoscopic ultrasonography images of the esophagus. Enodscopic ultrasonography images were shown. **a**-**b**: the mild(**a**) and serious(**b**) peri-ECV(arrow), **c**: serious para-ECV(arrow), **d**: PFV(arrow). The diameter of varicose vein was also measured. Figure 2e from Gut Liver 2017;11:843–851

In the course of EUP, the endoscopy used for EUP was GIF-CV290 (Olympus, Japan). A EU-ME1 Ultrasound endoscopy host with UM-3R,20-MHz catheter probe (Olympus, Japan) was also used. EUP was performed in one month after variceal eradication confirmed by endoscopy. EUP was not performed again during follow-up. The criteria for the EUP diagnosis were as follows: the multilayer structure stratification of the esophageal wall can be clearly visualized, including mucosa layer, mucosal muscle layer, submucosal layer, intrinsic muscle layer, and outer layer. The varicose veins around the esophagus aren’t connected to the intrinsic muscle layer, which is defined as para-esophageal collateral veins (para-ECVs); varicose veins is located in submucosal layer and no link with the intrinsic muscle layer, which is defined as peri-esophageal collateral veins (peri-ECVs), Penetrating the intrinsic muscle layer, The vein connects para-ECVs and submucosal vein, which is defined as perforating veins (PFV). The diameter of varicose vein was also measured. The results were recorded on EUP images, which were independently reviewed and interpreted by two senior endoscopists to reduce bias. The EUP images were shown in Fig. 2a-d.

### CT angiography

**Fig. 3:**
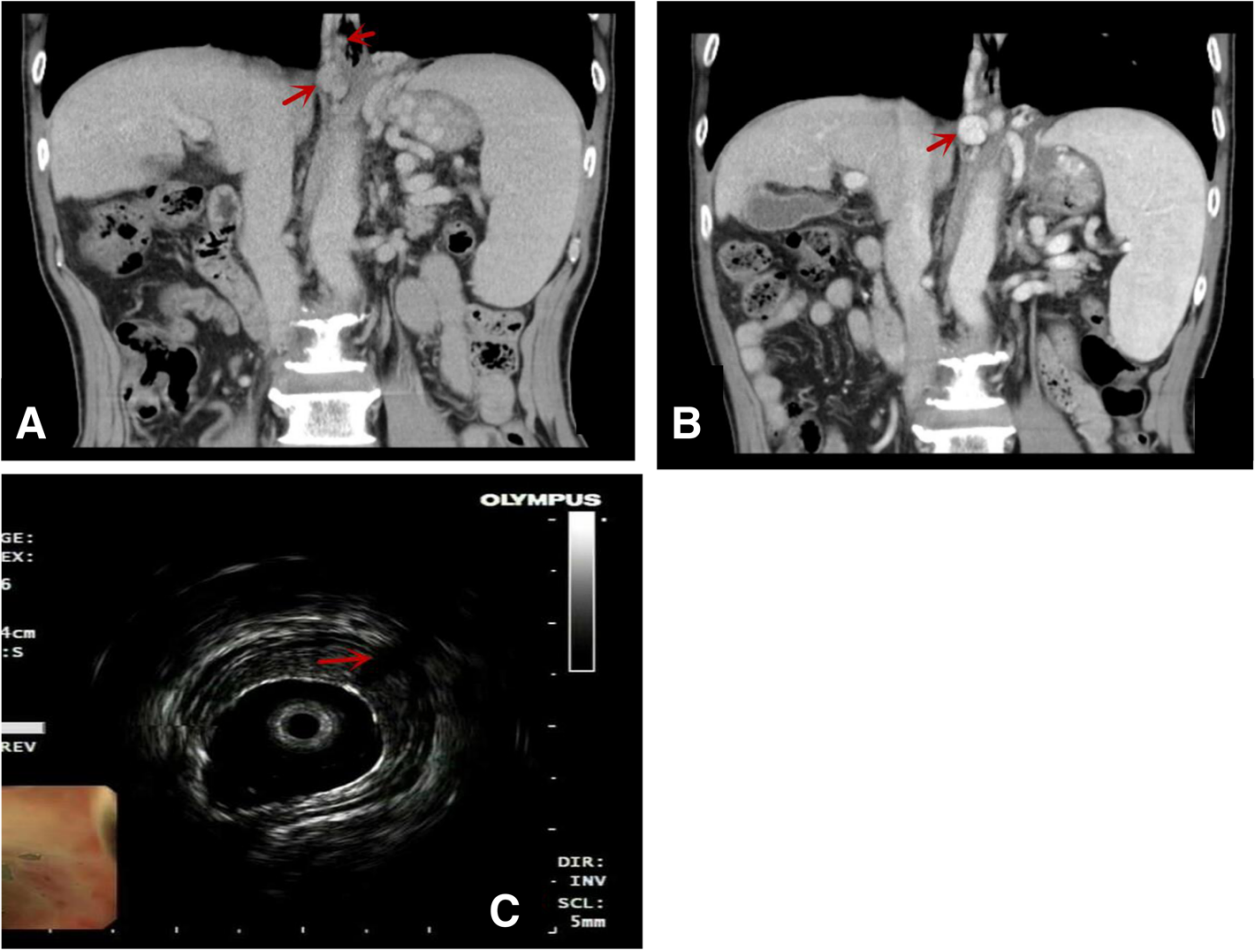
Comparison of CT vascular images before and after endoscopic treatment. **a**: Before the endoscopic treatment, varicose veins and esophageal varices near the esophagus can be seen. **b**: After endoscopic treatment, esophageal varices disappear, and varicose veins around the esophagus still exist. It is not well detected the perforating veins (PFV),but EUS can be shown(**c**:arrow)

4-phase multidetector computed tomography (CT) scan (GE HISPEED DXI; GE Company) with three-dimensional vascular reconstruction for the liver was routinely performed. The portal thrombosis, spontaneous spleno-renal shunts and portosystemic collateral veins were observed by 4-phase multidetector CT in the patients (Fig. 3a-c).

### Clinical and laboratory data

Symptoms, signs, and adverse events were recorded in the medical record. Serum markers for hepatitis B and C viruses were detected by electrochemiluminescence immunoassay (Roche E170 modular immunoassay analyzer, Roche Diagnostics, Mannheim, Germany). Serum biomarkers for the liver and renal function, including serum alanine aminotransferase (ALT), aspartate aminotransferase (AST), total bilirubin (TBIL), albumin, creatinine, and urea nitrogen, were measured on an automatic biochemical analyzer (AU5400, Olympus Company, Tokyo, Japan). Child-Pugh scores was calculated^11^; MELD (Model for-stage liver disease) is 3.8 × ln[TBiL(mg/dL)] + 11.2 × ln(INR) + 9.6 × ln[creatinine (mg/d1) + 6.4 × (cause of disease: biliary or alcoholic 0; other 1).

### The end-points and follow-up protocol

During the follow-up period, endoscopy and clinical data were collected once every six months, while CT images were reviewed once a year. The endpoints of follow-up were recurrence of esophageal varices, or rebleeding from esophageal varices or follow-ups to 3 years, liver transplantion or TIPS, and death. The criteria of recurrence of esophageal varices were defined as moderate esophageal varices, or mild varicose vein with red sign needed to be treated by endoscopy (Fig. 1d-e). The cirrhotic etiology and complications of portal hypertension were treated following the guidelines. Non-selective beta-blockers, however, were not used during follow-up period. Observation period was defined as the time interval between the end of the endoscopic treatment and the time of the endpoint.

### Statistical analysis

IBM SPSS 22 statistical software was used to statistical analysis. The quantitative data were presented as mean ± standard deviation (SD). The student t test and ANOVA for unpaired data were applied to compare differences between groups. The qualitative data were analyzed by the chi-square test or the Fisher’s exact probability test. Multivariate linear regression analysis was conducted to evaluate the correlation between multiple variables. The Kaplan–Meier estimator and log-rank test was used to analyze the survival. A *P* value less than 0.05 (two-way) was considered statistically significant.

## Results

### Demographic and clinical characteristics

The demographic and clinical characteristics of the study subjects are summarized in Table 1. The patients aged 28–75 years old, with 100 males and 53 females. Of these, 54 patients were treated with EVL, 80 patients were given EIS, and 19 cases received EVL and EIS in combination. The mean course of endoscopic treatment was 2.7. Among the 153 patients with varices in the esophagus, a majority of the patients (134 cases) had liver cirrhosis alone, while 19 patients had liver cirrhosis and cancer. The etiological factors of liver cirrhosis were also presented in Table 1.

**Table 1:** The demographic and clinical characteristics of the study patients

Variable		
Age(year)	All cases	55.2 ± 11.9(28–75)
Gender(*n*, %)	Male	100, 65.4
	Female	53, 34.6
Etiology of cirrhosis(*n*, %)		
	hepatitis B	100, 65.4
	hepatitis C	11, 7.2
	alcoholic liver disease	19, 12.4
	nonalcoholic fatty liver disease	10, 6.5
	autoimmune liver disease	10, 6.5
	unknown causes	3, 2.0
Cirrhosis with liver cancer(*n*,%)	Yes	19, 12.4
	NO	134, 87.6
Child-Pugh class (*n*,%)	A	96, 62.7
	B	43, 28.1
	C	14, 9.2
Endoscopic treatment (*n*,%)	EVL	54, 35.3
	EIS	80, 52.3
	EVL + EIS	19, 12.4
Recurrence of EV (*n*,%)	within 1 year	89, 58.2
	1–3 years	54, 35.3
Mean time of recurrence (months)	From eradication to recurrence	13.4

**Fig. 4:**
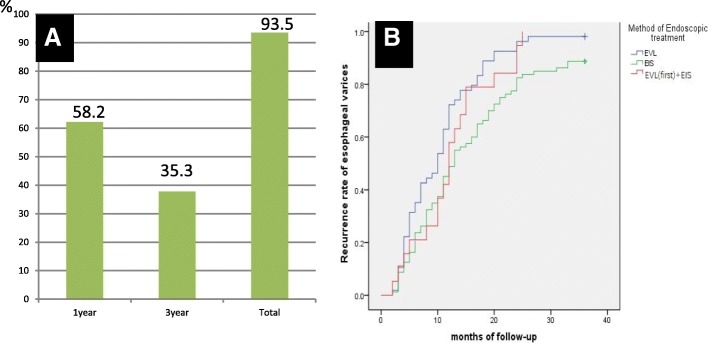
The recurrence rate of esophageal varices was shown during the follow-up period. The total recurrent esophageal varice was 93.5%. It revealed 58.2%, 35.3% within 1, 3 years, respectively, following the endoscopic esophageal varice eradication(**a**). The patients in the EVL group showed significantly shorter time of recurrence, compared with the EIS group as well as EIS + EVL group (**b**)

Notably, during the 3 years follow-up period, recurrence of esophageal varices occurred as high as 93.5% (143/153) patients, whereas considerably low non-recurrence rate at 6.5% (10/153) was observed after endoscopic eradication esophageal varices. The recurrent esophageal varice revealed 58.2%, 35.3% within 1, 3 years, respectively, following the endoscopic esophageal varice eradication (Fig. 4a). The mean time of recurrence of esophageal varices was 13.4 months.

### Risk factors associated with recurrence of esophageal varices

The risk factors in relation to recurrence of varices in 1 year, and 1–3 years following the endoscopic esophageal varice eradication were shown in Table 2. The high Child-Pugh score, large peri-ECVs, PFVs, and EVL, were independently risk factor identified to correlate with the recurrence of esophageal varices following the endoscopic treatment for esophageal varicel eradication. Furthermore, the univariate logistic regression analysis revealed that EVL (OR 0.23, 95% CI 0.08–0.71, *p* < 0.01), Child-Pugh score (OR 3.32, 95% CI 1.31–35.35, *p* < 0.05), large peri-ECVs (OR 4.56, 95% CI 2.17–9.58, *p* < 0.0001), and existence of PFV (OR 2.14, 95% CI 1.44–3.16, *p* < 0.001), were significantly associated with the recurrence of esophageal varices. Of these factors, the large peri-ECVs and PFV showed better ability to predict esophageal variceal recurrence. When the cut-off value of peri-ECVs was 3.5 mm, the specificity of prediction 1-year variceal recurrence was 86% and the sensitivity was 45%. The diameter of para-ECV(OR 0.99, 95% CI 0.79–1.25, *P* = 0.98)was not related to recurrence of esophageal following the endoscopic esophageal varice eradication.

**Table 2:** The risk factors of recurrence after esophageal variceal eradication by endoscopic therapies

	Within 1 year recurrence group (*n* = 89)	1–3 years recurrence(*n* = 54)	Without variceal recurrence group(*n* = 10)	*P* value
Gender				
Male(*n*,%)	58, 65.2	36, 66.7	6, 60	0.91
Female(*n*,%)	31, 34.8	18, 33.3	4, 40	
Endoscopic treatment				0.02
EVL(*n*,%)	39, 43.8	14, 25.9	1, 10	
EIS(*n*,%)	39, 43.8	32, 59.3	9, 90	
EVL + EIS(*n*,%)	11, 12.4	8, 14.8	0, 0	
Variceal diameter(cm)	1.22 ± 0.31	1.20 ± 0.3	0.9 ± 0.2	0.03
Red signs before treatment(Y/N)	68/21	36/18	5/5	0.14
Gastric varices(Y/N)	26/63	13/41	0/10	0.13
Diameter of peri-ECV(mm)	3.7 ± 1.4	2.5 ± 1.1	1.7 ± 0.9	0.0
Existence or not of PFV (Y/N)	57/32	2/52	0/10	0.0
Diameter of PFV(mm)	1.9 ± 1.6	0.1 ± 0.4	0	0.0
Diameter of para-ECV(mm)	6.5 ± 2.2	5.9 ± 1.9	6.9 ± 3.7	0.28
Primary/secondary preventions(n)	15/74	13/41	3/7	0.44
Child-Pugh grade(*n*,%)				0.04
A	49, 55.1	39, 72.2	8, 80	
B	27, 30.3	14, 25.9	2, 20	
C	13, 14.6	1, 1.9	0, 0	
Child-Pugh score	7.3 ± 2.1	6.5 ± 1.4	6.4 ± 0.9	0.025
MELD score	7.1 ± 7.8	6.9 ± 4.9	5.3 ± 2.0	0.72
CT angiography				
Veins around the esophagus(Y/N)	81/8	50/4	9/1	0.93
Veins around fundus (Y/N)	63/26	38/16	6/4	0.77
Other collateral vein(Y/N)	12/77	11/43	2/8	0.53
Portal vein embolus(Y/N)	25/64	10/44	3/7	0.41
With liver cancer/cirrhosis	8/81	9/45	2/8	0.31
Etiology of cirrhosis(*n*, %)				0.28
hepatitis B	60, 67.4	33, 61.1	7, 70	
hepatitis C	2, 2.2	8, 14.8	1, 10	
alcoholic liver disease	14, 15.7	4, 7.4	1, 10	
nonalcoholic fatty liver disease	6, 6.8	3, 5.6	1, 10	
autoimmune liver disease	6, 6.8	4, 7.4	0, 0	
unknown causes	1, 1.1	2, 3.7	0, 0	

### Effects of different endoscopic treatments on esophageal varicel recurrence

Next, we examined effects of EVL, EIS, and EVL plus EIS on the recurrence of varices in the esophagus (Fig. 4b). The patients in the EVL group showed significantly shorter time of recurrence (10 months), compared with the EIS group (13 months) as well as EIS + EVL group (12 months) (*P* < 0.05). However, a less courses were found in the EVL group (mean 1.9 courses) compared to the EIS group (mean 3.2 courses) and EVL plus EIS group (mean 3.4 courses) (*P* < 0.05). Also, we found that nine patients in the EIS group developed esophageal stenosis, which was 11.2% incidence rate, whereas none of patients was found to have this complication in the EVL group and EVL plus EIS group (P < 0.05) (Table 3). Moreover, the absence of PFV was found in a significant smaller proportion of patients in the EVL group (25/54, 46.3%), in contrast to that in the EIS group (56/80, 70%) and the EVL plus EIS group (13/19, 68.4%) (P < 0.05).

**Table 3:** The effects of endoscopic therapies on variceal recurrence after esophageal variceal eradication

	EVL group (*n* = 54)	EIS group (*n* = 80)	EVL + EIS group (*n* = 19)	*P* value
Course number of endoscopic treatment course to variceal eradication	1.9 ± 0.7	3.2 ± 1.2^a^	3.4 ± 1.6^a^	0.00
Median time of variceal recurrence(month)	10	13^a^	12	0.01
EUS findings				
diameter of peri-ECV(mm)	2.9 ± 1.4	3.4 ± 1.5	2.9 ± 1.1	0.14
diameter of PFV(mm)	1.44 ± 1.5	0.9 ± 1.5	1.0 ± 1.3	0.15
existence or not of PFV(Y/N,n)	29/25	24/56^a^	6/13	0.01
diameter of para-ECV(mm)	6.2 ± 2.1	6.3 ± 2.3	6.3 ± 2.4	0.83
Esophageal stenosis(Y/N,n)	0/54	9/71^a^	0/19	0.01
Child-Pugh grade(n,%)				0.21
A	29, 53.7	57, 71.3	10, 52.6	
B	20, 37.0	16, 20	7, 36.9	
C	5, 9.3	7, 8.7	2, 10.5	
Child-Pugh score	7.2 ± 2.1	6.7 ± 1.7	7.2 ± 1.9	0.36
Portal vein embolus(Y/N, n)	10/44	24/56	4/15	0.29
With liver cancer (n,%)	7, 13.0	9, 11.3	3,15.8	0.85
Unknown causes for cirrhosis	0	2	1	

^a^Compared with EVL group

### Comparison of CT angiography and EUP findings

CT angiography and EUP findings were shown in Table 4. EUP was more effective in the identification of para-EVS than CT angiograph (*P* < 0.001). However, CT angiography was unable to show peri-ECV and PFV. The visible portosystemic collateral veins, including spontaneous spleno-renal shunts or gastric-renal shunts, were not observed before endoscopic therapies in this cohort.

**Table 4:** Comparision between CT angiography and EUP findings

		CT angiography	
		para-ECVs (negative)	para-ECVs (positive)
EUP findings	para-ECV(negative)*n*,%	4, 2.6	0, 0
	para-ECV(positive)*n*,%	9, 5.9	140, 91.5

## Discussions

Hemorrhage of esophageal varices has been sought to be a serious complication in liver cirrhotic patients with portal hypertension, accounting for 20–40% mortality of cirrhotic patients with EVB within 6 months [12, 13]. Currently, endoscopic treatment is recommended as the first line therapy according to guidelines [2, 14, 15]. The key problems were the high recurrence of esophageal varices after the endoscopic esophageal variceal eradication therapies [16, 17]. However, up to now, it remains unclear about the risk factors associated with the recurrence of esophageal varices after varices were eradicated by endoscopic treatment. This large cohort prospective study has the following main novel findings: (1) A majority of the patients, accounting for 93% of the study subjects, developed recurrent esophageal varices within 3 years following variceal eradication; (2) The patients who underwent EVL were more likely to have recurrent esophageal varices compared to those with other endoscopic treatments; (3) high Child-Pugh score, large peri-ECV, and existence of PFV were identified as independent risk factors significantly correlated with the recurrence of esophageal varices; (4) The peri-ECV and PFV had good ability to predict recurrence of esophageal varices.

CT angiography and EUS were previously used to evaluate the effectiveness of endoscopic therapy for eradication of esophageal varices^.^, as it allows to clearly show diameter of hepatic portal vein and its collateral circulation [18, 19]. It has also been reported that a number of factors were related with esophageal varices bleeding, including the diameter of esophageal varices, red sign, Child-Pugh score, model for end-stage liver disease score(MELD), diameter of portal vein, portal vein embolus, hepatic venous pressure gradient (HVPG) [20]. In fact, the effect of above-mentioned factors, spontaneous spleno-renal shunts or portosystemic collateral veins in liver cirrhosis, on variceal recurrence are still not fully understood [21]. In this study, we confirmed that the C-P classification was associated with variceal recurrences. EUS and CT angiography are usually allowed to be directly observed collateral vascular structures and evaluate the vascular networks connected to esophageal varices In this study, EUP was also applied to probe the blood vessels around the wall of the esophagus, which appeared to be more effective in examination of para-ECVs, peri-ECVs and PFV, whereas CT angiography was unable to detect peri-ECVs and PFV. We also identified that high Child-Pugh score, method of endoscopic treatment were related with esophageal variceal recurrence, whereas the portal vein embolus had no effect on recurrence. There is a possibility that the reduced HVPG by collateral circulation accompanied by reduced recurrences of varicose veins, especially para-ECVs or spontaneous spleno-renal shunts or gastric-renal shunts and portosystemic collateral veins. In this study, however, peri-ECVs and PFV detected by EUP was significantly associated with the recurrence of esophageal varices. Some clinical studies were reporteded that patients who experienced EV recurrence after EVL were more likely to have severe-grade perforating veins before treatment than those without recurrence, severe peri-ECVs and multiple peri-ECVs were significantly associated with the risk of variceal recurrence, but not with para-ECVs or perforating vein [9, 22, 23]. In patients with portal hypertension, in fact, after esophageal varices were eradicated, other collateral circulation veins needed to be established, such as para-ECVs. In this study, no difference of para-ECVs were observed in both recurrent and non recurrent patients. Thus, the key action to preventing esophageal variceal recurrence could be the closure of PFV and peri-ECVs. Notably, in this study, the rate of closed peri-ECV was 46.3, 70.0% and 69.0.% respectively in EVL, EIS and EVL plus EIS group, which may contribute to the recurrence of esophageal varies. It was worthwhile to note in our study that there were significant differences in the recurrence time of esophageal varices following EVL and EIS. The median recurrence times in EVL group (10 months) was significantly shorter than that in the EIS group (13 months), and median recurrence months of EVL plus EIS group (12 months). Although EIS had a longer time of esophageal variceal eradication, the incidence of complications was relatively higher. The incidence rate of esophageal stenosis in the EIS group was 11.3%, whereas non of the EVL and EVL plus EIS group.. Compared with EIS, EVL has shorter recurrent time, but has less severe complication, for which EVL could be considered to safer than EIS. In addition, in comparison with MELD score, Child–Pugh score was more likely to be associated with the recurrence of esophageal varices. HVPG has been sought to be an ideal indicator to reflect portal hypertension, but some studies showed that there was slightly alteration before and after EVL, EIS treatment [24, 25].

Our study has limitations, including non-randomized design, lack of endoscopic ultrasonography before endoscopic treatment. The effect of beta-blockers, sunch as carvedilol or propranolol, combined with EVL or EIS on variceal recurrence deserves further clinical study.

## Conclusions

EUP sonography is useful for the evaluation esophageal varices recurrence after variceal eradication by endoscopic therapies. Our results have demonstrated that the high Child-pugh score, large para-ECV, and PFV are independent risk factors in the prediction of esophageal varices recurrence after variceal eradication by endoscopic therapies. The identified risk factors hold potential for clinical application in the future.
